# Viral Metagenomics on Cerebrospinal Fluid

**DOI:** 10.3390/genes10050332

**Published:** 2019-04-30

**Authors:** Arthur W. D. Edridge, Martin Deijs, Ingeborg E. van Zeggeren, Cormac M. Kinsella, Maarten F. Jebbink, Margreet Bakker, Diederik van de Beek, Matthijs C. Brouwer, Lia van der Hoek

**Affiliations:** 1 Laboratory of Experimental Virology, Department of Medical Microbiology, Amsterdam UMC, University of Amsterdam, 1105 AZ Amsterdam, The Netherlands; a.w.edridge@amc.uva.nl (A.W.D.E.); m.deijs@amc.uva.nl (M.D.); c.m.kinsella@amc.uva.nl (C.M.K.); m.f.jebbink@amc.uva.nl (M.F.J.); m.e.bakker@amc.uva.nl (M.B.); 2 Department of Neurology, Amsterdam UMC, University of Amsterdam, Amsterdam Neuroscience, 1105 AZ Amsterdam, The Netherlands; i.e.vanzeggeren@amc.uva.nl (I.E.v.Z.); d.vandebeek@amc.uva.nl (D.v.d.B.); m.c.brouwer@amc.uva.nl (M.C.B.)

**Keywords:** metagenomics, viromics, metaviromics, virus, CNS infection, cerebrospinal fluid, next-generation sequencing, VIDISCA-NGS, encephalitis

## Abstract

Identifying the causative pathogen in central nervous system (CNS) infections is crucial for patient management and prognosis. Many viruses can cause CNS infections, yet screening for each individually is costly and time-consuming. Most metagenomic assays can theoretically detect all pathogens, but often fail to detect viruses because of their small genome and low viral load. Viral metagenomics overcomes this by enrichment of the viral genomic content in a sample. VIDISCA-NGS is one of the available workflows for viral metagenomics, which requires only a small input volume and allows multiplexing of multiple samples per run. The performance of VIDISCA-NGS was tested on 45 cerebrospinal fluid (CSF) samples from patients with suspected CNS infections in which a virus was identified and quantified by polymerase chain reaction. Eighteen were positive for an RNA virus, and 34 for a herpesvirus. VIDISCA-NGS detected all RNA viruses with a viral load >2 × 10^4^ RNA copies/mL (n = 6) and 8 of 12 of the remaining low load samples. Only one herpesvirus was identified by VIDISCA-NGS, however, when withholding a DNase treatment, 11 of 18 samples with a herpesvirus load >10^4^ DNA copies/mL were detected. Our results indicate that VIDISCA-NGS has the capacity to detect low load RNA viruses in CSF. Herpesvirus DNA in clinical samples is probably non-encapsidated and therefore difficult to detect by VIDISCA-NGS.

## 1. Introduction

For patients with a suspected central nervous system (CNS) infection, rapid and accurate diagnosis is vital to determine treatment and improve prognosis [1]. The differential diagnosis of such patients includes infectious etiologies, of which viruses are the most common [2], but also non-infectious etiologies, such as auto-immune diseases [3]. Nonetheless, in more than half of cases, the cause remains unknown [4]. Identification of a virus can aid in patient management as it may initiate specific antiviral treatment, or cease or prevent ineffective antiviral, antibiotic, and/or immunosuppressive treatments, which all have potential harmful side effects. For example, when differentiating between an auto-immune and viral origin, immune suppression could lead to deleterious outcomes when caused by an unidentified virus [5].

During the last two decades, conventional diagnostics for viral CNS infections have shifted from non-specific culturing techniques towards highly-specific viral nucleic acid amplification tests, like quantitative polymerase chain reaction (qPCR), or the detection of host-mediated antibody production to the virus (e.g., ELISA). Although these latter assays have greatly increased diagnostic sensitivity, a limitation is that they only target an individual virus or a subset of related viruses. The number of viruses that have been associated with CNS infections currently comprises more than 100 [6], with several more discovered in the last decade [7,8,9,10]. Consequently, a comprehensive diagnostic panel would include many specific tests. Since this is unachievable for routine diagnostics, only a small selection of viruses commonly associated with CNS infections are included in most diagnostic panels (e.g., herpes simplex virus 1/2, enteroviruses, and parechoviruses). Other pathogens are usually not examined, or are tested for at a later stage of the disease, by which time irreversible pathology could have occurred.

Metagenomics is a recent and promising development in microbiology, which is theoretically able to detect all viruses, including known, unexpected, and novel species [5]. The sensitivity of such assays is generally determined by three factors: (1) The concentration of viruses in a clinical sample, (2) the amount of background (competing) RNA and DNA, and (3) the sequencing depth. Generally, metagenomics assays are poor or unable to detect viruses in a clinical specimen because of the low viral load relative to the high concentration of background RNA and DNA. To overcome this, viral metagenomic assays enrich the viral content of a sample. Virus discovery cDNA-AFLP (amplified fragment length polymorphism) next-generation sequencing (VIDISCA-NGS) is one of the available assays for viral metagenomics. Characteristic for VIDISCA-NGS is the fragmentation of ds(c)DNA, which is done using a frequent-cutting restriction enzyme, and thus different from the random shearing, random PCR amplification, or transposon-based shearing techniques used in most viral metagenomic assays [11,12]. The method was first described with the discovery of human coronavirus NL63 [13], and since has discovered and detected a wide range of viruses in various sample types [14,15,16,17,18]. VIDISCA-NGS could be an ideal tool for the broad range detection of viruses in cerebrospinal fluid (CSF).

CSF is a distinct bodily fluid containing a relatively low number of host cells. Even with mild pleiocytosis, as seen during most viral CNS infections, CSF has a far lower cellular content than a similar volume of blood, respiratory, or fecal material. This low amount of background could influence NGS results in two ways: (1) It may decrease the nucleic acid extraction yield if the total nucleic acid content is too low, or (2) it may be beneficial, as proportionally less sequence space is taken by competing background RNA or DNA. Considering the potential benefit viral metagenomics may have for future viral diagnostics in encephalitis, we determined the capability of VIDISCA-NGS to detect viruses in CSF samples from patients with suspected CNS infections.

## 2. Materials and Methods

CSF samples which previously tested positive by viral qPCR were selected from two biobanks of the departments of medical microbiology and neurology of the Amsterdam UMC (location AMC). An HIV-1 qPCR was performed using the RealTime HIV-1 Viral Load Assay (Abbott Molecular, Abbott Park, IL, USA), the other viruses were tested by in-house qPCRs using previously published methods [19]. The first sample set consisted of anonymized leftover CSF samples (n = 27), sent in from patients with suspected CNS infection. The second set of CSF samples (n = 18) were selected from a clinical study on the etiology of encephalitis and meningitis in adult patients [2]. The study was approved by the medical ethics committee of the Academic Medical Centre, Amsterdam, The Netherlands (reference number 2014_290). All samples had a quantifiable viral load and were stored at −80 °C until library preparation for VIDISCA-NGS.

VIDISCA library preparation was performed as previously described [9,17]. Briefly, CSF samples were centrifuged and the supernatant was treated with TURBO™ DNase (Thermo Fisher Scientific, Waltham, MA, USA) to remove naked chromosomal or bacterial DNA. Nucleic acids were extracted using the Boom method [20], followed by reverse transcription with non-ribosomal random hexamers [21] and second strand synthesis. DNA was digested with MseI (T^TAA; New England Biolabs, Ipswich, MA, USA) and ligated to adapters containing a sample identifier sequence. During the fragmentation in VIDISCA, the sample cannot be “over-digested” as fragmentation relies entirely on the presence of restriction enzyme recognition sites and not on the duration of fragmentation. Ligation to adaptors leads to loss of the restriction enzyme recognition site (after ligation to an adaptor the sequence is TTAT) whereas ligation to another DNA fragment will restore it, allowing re-digestion. Next, size selection with AMPure XP beads (Beckman Coulter, Brea, CA, USA) was performed to remove small DNA fragments prior to a 28-cycle PCR using adaptor-annealing primers. Small and large size selection was performed with AMPure XP beads to select DNA-strands with a length ranging between 100 and 400 nucleotides. Libraries were analyzed using the Bioanalyzer (High Sensitivity Kit, Agilent Genomics, Santa Clara, CA, USA) and Qubit (dsDNA HS Assay Kit, Thermo Fisher Scientific) instruments to quantify DNA length and concentration, respectively. Seventy sample libraries were pooled at the equimolar concentration. The current number of 70 samples was chosen because this has worked for other sample types (non-CSF) [16,17]. In total, 50 pmol DNA of the pool was clonally amplified on beads using the Ion Chef System (Thermo Fisher Scientific) and sequencing was performed on the Ion PGM™ System (Thermo Fisher Scientific) with the ION 316 Chip (400 bp read length and 2 million sequences per run). The method for the DNase-free VIDISCA library preparation omitted the TURBO™ DNase step.

All VIDISCA-NGS reads with a minimum length of 45 nucleotides were translated into protein sequences and aligned to a local database of the NCBI eukaryotic viral Identical Protein Groups (downloaded March 2018) using UBLAST [22], the VIDISCA bioinformatics workflow [23], and an online metagenomic profiler (Taxonomer) [24] for identification of probable viral reads and background sequence classification. Probable viral reads were subsequently confirmed when the original VIDISCA-NGS read could be aligned to a reference sequence of the virus with a nucleotide identity of at least 80% using CodonCode Aligner (version 6.0.2). Each alignment was manually inspected for confirmation. Samples were considered VIDISCA-NGS positive when at least one VIDISCA-NGS read could be identified. The number of reads aligned to a reference sequence in CodonCode Aligner was taken as the number of viral reads per sample. Analysis by VIDISCA-NGS was performed blind to qPCR results to avoid biased analysis. All statistical analyses were performed in R (version 3.5.1), and graphs were plotted using R package ggplot2 (version 3.1.0).

## 3. Results

### 3.1. Sample Description and qPCR Results

Forty-five CSF samples from patients with a suspected CNS infection were examined. Samples had been tested by routine diagnostic for enterovirus, human immunodeficiency virus 1 (HIV-1, in case the patients were HIV-1 seropositive), parechovirus, and herpesviruses (herpes simplex virus 1 and 2 (HSV-1/2), varicella-zoster virus (VZV), Epstein-Barr virus (EBV), cytomegalovirus (CMV), and human herpesvirus 7 (HHV-7). The CSF samples contained either a single virus (n = 36) or multiple viruses (n = 9), and tested positive for HIV-1 (n = 10), enterovirus (n = 8), HSV-1/2 (n = 14), VZV (n = 8), EBV (n = 12), CMV (n = 2), and HHV-7 (n = 2). All details concerning the qPCR-results, viral loads, total sequence reads obtained via VIDISCA-NGS, and number of viral sequences are available in the Supplementary Table S1.

### 3.2. RNA Virus Detection by VIDISCA-NGS

**Figure 1:**
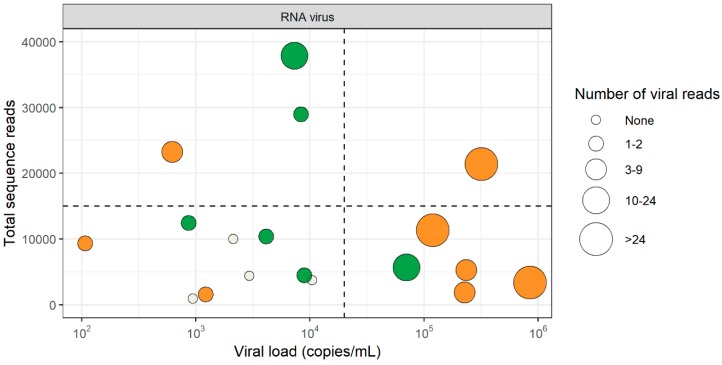
Detection of RNA viruses by virus discovery cDNA-AFLP (amplified fragment length polymorphism) next-generation sequencing (VIDISCA-NGS) in cerebrospinal fluid (CSF). Green dots: samples that were positive by VIDISCA-NGS for enterovirus, orange dots: samples that were positive by VIDISCA-NGS for HIV-1, white dots: samples that were negative by VIDISCA-NGS. The size of the dots corresponds to the number of viral reads. On the x-axis, the viral load in CSF is displayed; on the y-axis, the total number of sequence reads. Samples are divided into segments by a horizontal line at 15,000 reads and a vertical line at 2 × 10^4^ RNA copies/mL.

Six samples were positive for enterovirus and eight for HIV-1 by VIDISCA-NGS, all of which were also qPCR positive (Figure 1). The RNA virus concentration in the VIDISCA-NGS positive samples ranged between 1.07 × 10^2^ RNA copies/mL and 8.64 × 10^5^ RNA copies/mL (median: 8.63 × 10^3^ RNA copies/mL). Two samples positive for enterovirus and two for HIV-1 by qPCR were missed by VIDISCA-NGS, with viral loads ranging from 9.40 × 10^2^ to 1.05 × 10^4^ RNA copies/mL (median 2.54 × 10^3^ RNA copies/mL).

**Figure 2:**
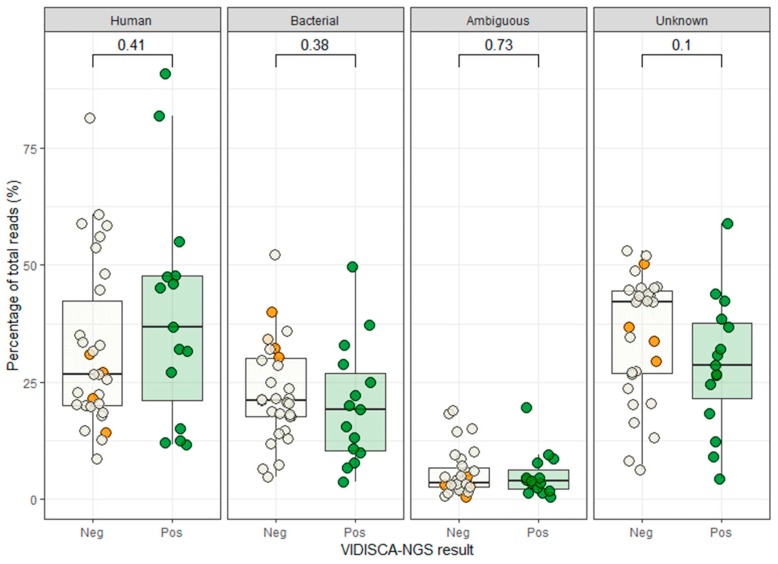
Background sequences in VIDISCA-NGS. Green dots: samples that were positive by VIDISCA-NGS, white dots: samples that were negative by VIDISCA-NGS, orange dots: the four samples containing an RNA virus not found by VIDISCA-NGS. On top the *p*-values are shown for the Mann-Whitney U test between the positive and negative VIDISCA-NGS samples. “Human” indicates human mitochondrial or genomic background, “Bacterial” indicates prokaryotic background, “Ambiguous” represents sequences with simultaneous hits to eukaryotes and prokaryotes, and “Unknown” are the sequences that do not match with any reference sequence.

To exclude that competition by background nucleic acids or other viruses might have hampered virus detection, we assessed whether co-infection by other pathogens or large quantities of the host genomic background had competed with viral sequences in the four samples that were negative in VIDISCA-NGS. The profile of the background sequences of the negative samples was similar to those of the positive samples, indicating that no major sequence competition was present (Figure 2). Next, we determined whether the sequencing depth of the four negative samples, in combination with the low viral load, may have been insufficient. All four missed samples had fewer than 10,000 sequence reads and had a viral load below 2 × 10^4^ copies/mL, as depicted in the lower left quadrant of Figure 1. Overall, this quadrant contained nine samples of which five were positive and four were negative by VIDISCA-NGS. The five positive samples had only one (n = 4) or two (n = 1) reads mapped to the detected RNA virus. These small numbers of viral reads suggest that such samples (with low viral load, combined with a low sequencing depth) were on the detection limit of VIDISCA-NGS. Samples with a similarly low viral load, but with a higher sequence depth (upper left quadrant of Figure 1), had, on average, more than 5 viral reads per sample. Moreover, a correlation between sequence depth and viral read number was seen for all samples below 10^4^ RNA copies/mL (rho = 0.64 *p* = 0.02, Spearman’s rank correlation test).

### 3.3. DNA Virus Detection by VIDISCA-NGS

Only one sample was VIDISCA-NGS positive for a herpesvirus (VZV), which was also qPCR positive at a concentration of 9.29 × 10^7^ DNA copies/mL. Among the samples that remained herpesvirus negative by VIDISCA-NGS, 33 were positive for at least one herpesvirus by qPCR (median: 9.01 × 10^3^, range: 5.28 × 10^3^–1.62 × 10^7^ DNA copies/mL). Because of the poor performance of VIDISCA-NGS, we hypothesized that our library preparation method, which uses a specific restriction enzyme, may have hampered herpesvirus detection. We examined the number of putative VIDISCA-NGS fragments (the number of unique genomic fragments that can theoretically be detected by VIDISCA-NGS based on the location of the Mse1 restriction enzyme recognition sites and resulting fragments lengths) in the human herpesvirus genomes. All human herpesviruses genomes have at least 16 putative VIDISCA fragments (Table 1). By comparison, the enterovirus and HIV-1 genomes produced a nearly equal number of fragments and were detected at a high success rate as described above.

Next, we hypothesized that the nuclease treatment may have hampered the detection of herpesvirus DNA. DNase treatment is done prior to nucleic acid extraction to remove naked chromosomal and bacterial DNA. It is assumed that viral genomic DNA is protected from DNase by the virus particle, however, if viral DNA is non-encapsidated, it will also be degraded. We therefore repeated the library preparation for all 45 CSF samples, now without a DNase treatment. 

### 3.4. Virus Detection by DNase-Free VIDISCA-NGS

**Figure 3:**
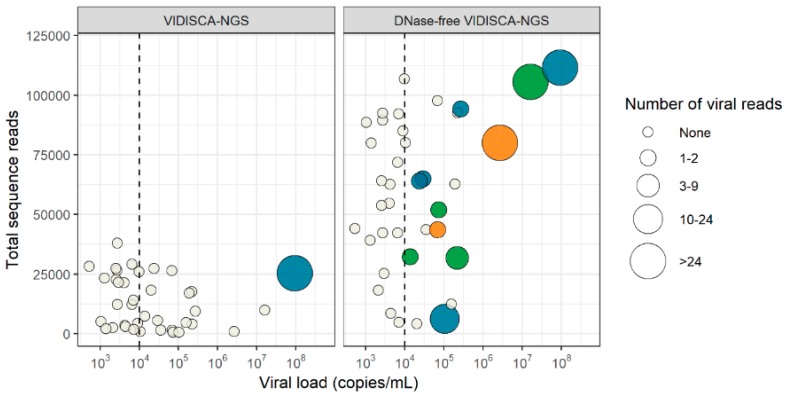
Detection of herpesviruses by VIDISCA-NGS in CSF. The results of regular VIDISCA-NGS are in the left panel, results of DNase-free VIDICSA-NGS are in the right panel. If a sample contained multiple viruses, multiple data points are displayed for each of the co-infecting viruses. A vertical line is drawn to separate samples above and below 10^4^ DNA copies/mL. Green dots: samples that were positive by VIDISCA-NGS for HSV-1/2, blue dots: samples that were positive by VIDISCA-NGS for VZV, orange dots: samples that were positive by VIDISCA-NGS for CMV, white dots: samples that were negative by VIDISCA-NGS. The size of the dots corresponds to the number of viral reads. On the x-axis, the viral load in CSF is displayed; on the y-axis, the total number of sequence reads.

With the DNase-free VIDISCA-NGS, only eight samples contained sequences of an RNA virus (six HIV-1 and two enterovirus) (Table 2), indicating that background DNA seriously hampered detection of RNA viruses. On the other hand, detection of herpesviruses greatly increased. Without a DNase treatment, 11 samples became VIDISCA-NGS positive: four for HSV-1/2, five for VZV, and two for CMV (Figure 3). The viral load of the nuclease-free VIDISCA-NGS herpesvirus positive samples was higher (median: 1.04 × 10^5^) than the negative samples (median: 4.42 × 10^3^, *p* = 0.00009, Mann Whitney U test). This association between the virus load and VIDISCA-detection became more visible when 10^4^ DNA copies/mL was taken as a threshold; 11 of 18 samples positive by qPCR with >10^4^ DNA copies/mL were also positive by VIDISCA-NGS, but none below.

### 3.5. Effect of a DNase Treatment on Virus Detection by VIDISCA-NGS

**Figure 4:**
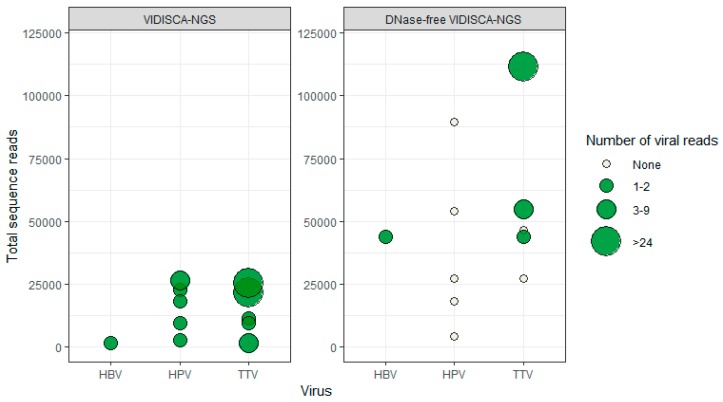
Effect of DNase on the detection of non-herpes DNA viruses by VIDISCA-NGS. On the x-axis, the viral species is displayed; on the y-axis, the total number of sequence reads. Left panel: Normal VIDISCA-NGS, right panel: DNase-free VIDSCA-NGS. Green dots: samples positive for the indicated virus, white dots: samples negative for the indicated virus. The size of the dots corresponds to the number of viral reads.

We identified several co-infecting DNA viruses (torque teno virus (TTV), n = 5; human papillomavirus (HPVs), n = 5; and hepatitis B virus (HBV), n = 1), which were not included in the routine diagnostics of the CSF samples, but were identified by VIDISCA-NGS (n = 11). Similar to the effects we observed for herpesvirus detection, we hypothesized that more non-herpes DNA viruses would be detected under the DNase-free condition. Surprisingly, no additional non-herpes DNA viruses were identified using the DNase-free method. On the contrary, of the 11 samples containing non-herpes DNA viruses detected by regular VIDISCA-NGS, only four samples were positive when excluding a DNase treatment (Figure 4).

**Figure 5:**
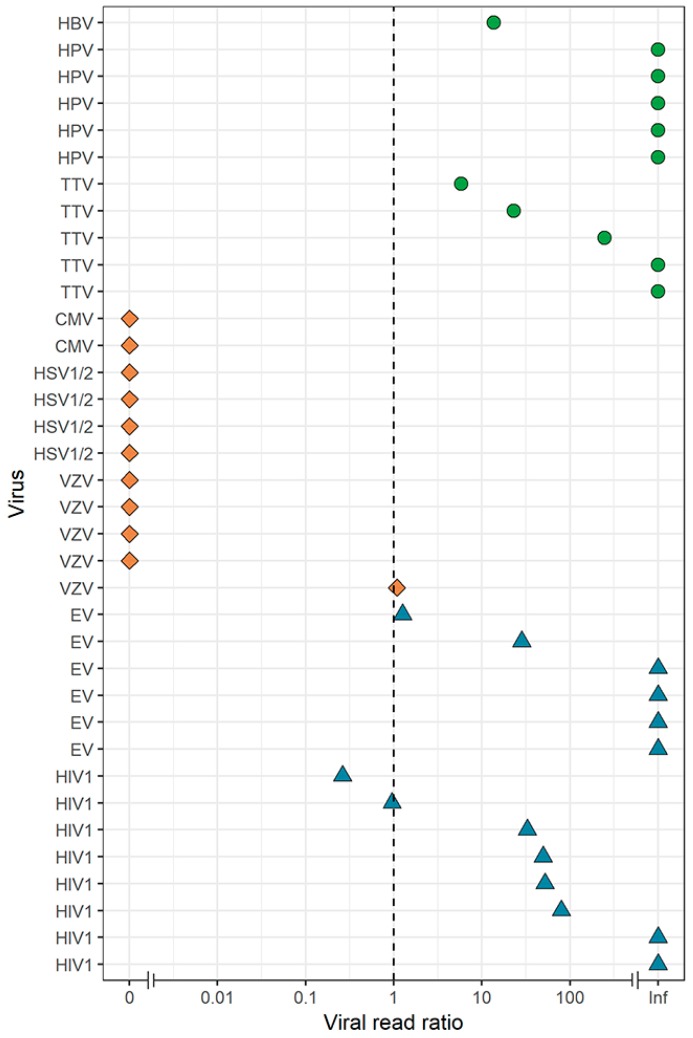
Effect of DNase on the detection of RNA and DNA viruses by VIDISCA-NGS in CSF. Viral read ratio (x-axis) is calculated as the ratio between the number of viral reads for samples with and without a DNase treatment, adjusted for the sequencing depth. Samples with a ratio >1 favor regular library preparation whereas samples with a ratio <1 favor a DNase-free treatment. Green dots: non-herpes DNA viruses, orange diamonds: herpesviruses, blue triangles: RNA viruses. On the y-axis, the viral species are displayed.

To assess the overall effect of a DNase treatment, we determined the ratio of viral reads, adjusted for sequencing depth, between the two treatment arms for all viruses identified by VIDISCA-NGS in this study (Figure 5). All herpesviruses had substantially more, or a roughly equal number of viral reads in the DNase-free condition. In contrast, the opposite was true for non-herpes DNA and RNA viruses.

## 4. Discussion

Metagenomic assays have the potential to benefit the diagnosis of CNS-infections. To this end, they need to meet certain prerequisites: Besides being broad—preferably detecting all viruses—an assay should be fast, sensitive, and affordable. VIDISCA-NGS is a unique method for viral metagenomics, which requires a relatively limited sequence depth and allows multiplexing, which reduces costs and runtime per sample [23]. As limited sequence depth, multiplexing, and speed may come at the expense of sensitivity, we evaluated the performance of VIDISCA-NGS on 45 clinical CSF samples containing viruses, quantified via conventional diagnostics (qPCR). VIDISCA-NGS detected an RNA virus in all medium to high viral load samples (>2 × 10^4^ RNA copies/mL) and most (67%) of the low viral load samples. One VIDICSA-NGS positive HIV-1 sample had only 1.07 × 10^2^ RNA copies/mL, demonstrating the capability to detect even very low load viruses.

Metagenomics has been used to detect novel or unexpected viruses in CSF in several studies [7,8,9,10], but only a limited number of studies have evaluated the performance. Two studies investigated the limit of detection using dilutions of spiked HIV-1 in CSF. One study used the Ribo-SPIA pipeline [25], the second used a tailor-made protocol, including Nextera, to fragment and amplify [26,27]. Both studies used >5 million reads per sample and found a limit of detection of ≈10^2^ RNA copies/mL for HIV-1, comparable to that of VIDISCA-NGS when 10,000 reads are used.

Besides the pathogens detected in the current study, VIDISCA-NGS has been able to detect a large number of other viruses, including members of the *Adenoviridae*, *Anelloviridae*, *Caliciviridae*, *Coronaviridae*, *Flaviviridae*, *Hepadnaviridae*, *Orthomyxoviridae*, *Paramyxoviridae*, *Parvoviridae*, *Peribunyaviridae*, *Picornaviridae*, *Pneumoviridae*, *Polyomaviridae*, and *Retroviridae*, in several types of clinical material (stool, serum, plasma, respiratory swabs) [14,15,16,17,18,28,29,30,31,32]. Thus, it is likely that VIDISCA-NGS is able to detect viruses from these families in CSF with similar sensitivities. However, our current findings now indicate there is one viral family difficult to detect with VIDISCA-NGS, namely the *Herpesviridae*. VIDISCA-NGS was only able to detect one high load herpesvirus (VZV, 9.29 × 10^7^ DNA copies/mL) out of 34 qPCR positive samples. We hypothesized that our nuclease treatment hindered herpesvirus detection, and omitting a DNase treatment indeed yielded an additional 10 samples that were positive for herpesvirus. Of the medium to high load herpesviruses (>10^4^ RNA copies/mL), DNase-free VIDISCA-NGS detected 61%.

The vulnerability of herpesviruses to DNase is not unexpected. Boom et al. found that CMV DNA in serum and plasma is highly fragmented and susceptible to DNases [33]. Similarly, Perlejewski et al. described a four-fold decrease in HSV-1 reads when using a DNase treatment for metagenomics on CSF [34]. Our study expands on this knowledge by showing that the vulnerability to DNase also applies to the other herpesviruses. This vulnerability signifies that the performance of metagenomic assays should not be evaluated on spiked samples. Herpesvirus culture harvests contain infectious virions with non-fragmented DNA [33,35], whereas herpesvirus in cell-free clinical material is non-infectious and, as mentioned above, contains highly fragmented DNA [33,36]. The only two studies that examined the performance of a metagenomics assay to detect herpesviruses used virus culture harvests, and found low limits of detection (≈10^1^ and 10^3^ DNA copies/mL for CMV and HSV-1, respectively) [25,26]. Caution should be taken to translate these findings to a clinical setting, as virus culture harvests are, especially for herpesviruses, not a correct representative of reality.

Herpesviruses have large DNA genomes and use rolling-circle amplification to produce head-to-tail concatamers of progeny virus [37]. During the lytic replication phase, large amounts of non-infective naked progeny virus are released from the cell and may enter the CSF if replication occurs in the CNS compartment. Because of the high genome copy number and the generally low DNase activity in CSF [38], degradation may take a significant amount of time. Naked herpesvirus DNA could thus persist for an extensive amount of time in CSF, even after the local infection has ceased. In theory, the persistence of naked DNA could also occur for other DNA viruses, such as HPV and TTV. These viruses use similar replication strategies to herpesviruses. The detection of these DNA viruses by VIDISCA-NGS was, however, not hampered by a DNase treatment (Figure 4), indicating that the viral DNA of these viruses was part of an intact virion.

Without amplification, the nucleic acid yield from CSF is generally too low for effective NGS library preparation for metagenomics [39]. For that reason, VIDISCA-NGS implements an amplification step to increase the number of viral genomic fragments from CSF. We previously found that viruses with a concentration of >10^4^ copies/mL were detected when 5000 sequence reads or more were generated per sample from nasopharyngeal swabs [17]. Since then, we have used this number as a threshold to ensure that a sufficient sequence depth was achieved for virus detection. Our current results suggest this threshold may have to be increased for CSF. All RNA virus samples missed by VIDISCA-NGS had fewer than 10,000 reads and a strong correlation between the sequencing depth and number of viral reads was observed. Increasing the sequence depth could therefore increase the detection of low load RNA viruses. As such, we recommend to generate 10,000 or more reads per sample.

In the current study, we multiplexed 70 samples per VIDISCA-run. While it is uncommon for a large number of patients with encephalitis to present at the same time, this method could be of substantial benefit in outbreaks [40] and research settings where large cohorts of patients have to be screened at the same time. Because the performance of VIDISCA-NGS remains lower than qPCR, especially for the detection of herpesviruses, VIDISCA-NGS cannot replace conventional diagnostics. Nonetheless, we suggest the use of standard VIDISCA-NGS (including a DNase) in parallel with conventional diagnostics, as this provides a cheap, low-input, and sensitive method to detect known, rare, and novel viruses in CSF.

## Figures and Tables

**Table 1:** Putative number of VIDISCA fragments per virus.

Virus	Fragments (n)
HSV-1	40 ^1^
HSV-2	16
VZV	352
EBV	129
CMV	137
HHV-7	473
Enterovirus	22
HIV-1	19

^1^ Number of putative VIDISCA fragments as determined by the number of genomic regions demarcated by two MseI restriction enzyme recognition sites with a length of 100 to 400 nucleotides. HSV-1: herpes simplex virus 1, HSV-2: herpes simplex virus 2, VZV: varicella-zoster virus, EBV: Epstein-Barr virus, CMV: cytomegalovirus, HHV-7: human herpes virus 7, HIV-1: human immunodeficiency virus 1.

**Table 2:** Performance of VIDISCA-NGS to detect viruses compared to quantitative polymerase chain reaction (qPCR) in CSF.

	VIDISCA-NGS	DNase-free VIDISCA-NGS
**RNA virus**		
Enterovirus	6/8 ^1^	2/8
HIV-1	8/10	6/10
Total	14/18	8/18
**Herpesvirus**		
HSV-1/2	0/14	4/14
VZV	1/8	5/8
EBV	0/12	0/12
CMV	0/2	2/2
HHV-7	0/2	0/2
Total	1/38	11/38

^1^ Results shown as: VIDISCA-NGS positives samples/qPCR positive samples.

## Supplementary Materials

The following are available online at https://www.mdpi.com/2073-4425/10/5/332/s1, Table S1: VIDISCA-NGS and qPCR data for all samples.
