# Supplementary material for: Viral Metagenomics on Cerebrospinal Fluid
Source: Genes (Basel). 2019 Apr 30;10(5):332. doi: 10.3390/genes10050332 (PMC6562652; doi:10.3390/genes10050332)
Supplement: Supplementary file 1 [file genes-10-00332-s001.pdf]

Table S1 – VIDISCA-NGS and qPCR data for all samples

Sample	Virus	qPCR result	Viral load (copies/mL)	Total seq reads[1]	DNase-free tot seq reads[2]	Viral reads[3]	DNase-free vir reads[4]	Human reads	Bacterial reads	Ambiguous reads	Unknown reads
1	HSV1/2	Pos	6520	12361	42257	0	0	1569	3526	1085	5371
1	HHV7	Pos	2740	12361	42257	0	0	1569	3526	1085	5371
2	HSV2	Pos	13800	7289	32308	0	1	1300	1375	1340	3199
3	VZV	Pos	268000	9442	94294	0	2	2513	1713	571	4607
4	HSV1	Pos	218000	17609	31925	0	7	5927	3785	542	6080
5	EV	Pos	4120	10408	84357	1	0	8512	704	192	955
6	VZV	Pos	528	28311	44136	0	0	17200	1837	1699	3741
7	HIV1	Pos	317895	21434	54646	77	6	10199	2158	1665	9075
7	EBV	Pos	4070	21434	54646	0	0	10199	2158	1665	9075
7	TTV	NT[5]		21434	54646	27	3	10199	2158	1665	9075
8	EBV	Pos	2570	22750	53860	0	0	1994	11879	568	6079
8	HPV	NT		22750	53860	1	0	1994	11879	568	6079
9	EBV	Pos	1050	5162	88644	0	0	3012	1086	142	844
10	HSV1/2	Pos	20300	18198	4285	0	0	3623	5393	604	8270
10	HPV	NT		18198	4285	1	0	3623	5393	604	8270
11	EBV	Pos	68300	26482	97739	0	0	15581	1253	5032	2181
12	HIV1	Pos	119445	11371	46613	39	2	3653	2535	532	4372
12	TTV	NT		11371	46613	2	0	3653	2535	532	4372
13	EV	Pos	862	12469	37485	1	0	6870	1927	165	3290
14	EBV	Pos	2710	26556	89554	0	0	5331	4695	1300	11296
14	HPV	NT		26556	89554	3	0	5331	4695	1300	11296
15	HIV1	Pos	1220	1593	43667	1	0	759	124	22	489
15	HPB	NT		1593	43667	1	2	759	124	22	489
15	CMV	Pos	69000	1593	43667	0	2	759	124	22	489
15	TTV	NT		1593	43667	9	1	759	124	22	489
15	EBV	Pos	35200	1593	43667	0	0	759	124	22	489
16	HSV1/2	Pos	9870	26014	106809	0	0	6925	3073	3742	10960
17	EBV	Pos	2110	2577	18265	0	0	579	643	110	1155
17	HBV	NT		2577	18265	1	0	579	643	110	1155
18	HSV1/2	Pos	224000	4051	92601	0	0	925	740	200	2149
19	HSV1	Pos	73000	666	52047	0	1	136	138	22	301
20	VZV	Pos	29300	5549	65009	0	1	1750	978	395	2335
21	HIV1	Pos	846080	3409	62677	38	14	395	852	85	2010
21	EBV	Pos	4280	3409	62677	0	0	395	852	85	2010
22	HIV1	Pos	107	9343	27134	1	0	2532	1797	806	4101
22	HPV	NT		9343	27134	2	0	2532	1797	806	4101
22	TTV	NT		9343	27134	2	0	2532	1797	806	4101
23	VZV	Pos	104000	680	6343	0	23	126	95	103	354
24	HSV1/2	Pos	190000	17045	62707	0	0	5605	3648	446	7233
25	HIV1	Pos	234220	5280	34480	8	1	2382	1061	205	1513
26	HSV1	Pos	16200000	9925	105692	0	44	2552	2030	937	4312
27	VZV	Pos	7000	14016	92184	0	0	7871	1821	1414	2842
28	HSV1/2	Pos	1410	2067	79948	0	0	924	489	58	563
29	HSV1/2	Pos	2940	21485	25351	0	0	3149	7710	599	9150
30	HIV1	Pos	620	23269	39097	7	45	21117	887	127	1047
30	EBV	Pos	1300	23269	39097	0	0	21117	887	127	1047
31	VZV	Pos	23700	27314	64126	0	1	22213	2029	221	1715
31	EBV	Pos	2540	27314	64126	0	0	22213	2029	221	1715
32	CMV	Pos	2670000	957	80067	0	146	136	290	30	482
32	EBV	Pos	10500	957	80067	0	0	136	290	30	482
32	HIV1	Pos	940	957	80067	0	0	136	290	30	482
33	VZV	Pos	9050	4388	85014	0	0	2357	944	162	898
34	HIV1	Pos	10485	3765	43946	0	0	1164	1284	22	1270
35	HSV2	Pos	157000	4693	12485	0	0	1641	1604	76	1274
36	HSV2	Pos	4420	3008	8618	0	0	612	966	59	1357
37	EV	Pos	70104	5714	103412	22	14	689	2841	264	1838
38	HIV1	Pos	225930	1869	4777	6	16	235	694	77	688
38	EBV	Pos	7190	1869	4777	0	0	235	694	77	688
39	VZV	Pos	92900000	25495	111578	25	101	8072	3344	5024	4673
39	TTV	NT		25495	111578	44	33	8072	3344	5024	4673
40	EV	Pos	2129	9982	61362	0	0	2708	3997	298	2943
41	HSV1/2	Pos	6520	29228	71830	0	0	14096	4312	435	6948
42	EV	Pos	2953	4386	7949	0	0	945	1413	207	1609
43	EV	Pos	8328	28999	97887	1	0	13367	3130	2765	7135
44	EV	Pos	7298	37912	92529	12	0	5761	10937	1303	4678
44	HHV7	Pos	2740	37912	92529	12	0	5761	10937	1303	4678
45	EV	Pos	8924	4518	14263	2	5	1662	1484	159	1205

[1]Total sequence reads per sample for VIDISCA-NGS
[2]Total sequence reads per sample for DNase-free VIDISCA-NGS
[3]Number of detected viral reads per virus by VIDISCA-NGS
[4]Number of detected viral reads per virus by DNase-free VIDISCA-NGS
[5]NT: Not tested
